# Fetal Heart Tracing Patterns and the Outcomes of Newborns With Meconium-Stained Amniotic Fluid

**DOI:** 10.7759/cureus.24545

**Published:** 2022-04-28

**Authors:** Mohammad Adnan, Janardhan Mydam, Joseph R Hageman, Lourdes Cohen

**Affiliations:** 1 Neonatology, Indiana University Health Ball Memorial Hospital, Muncie, USA; 2 Neonatology, John H. Stroger, Jr. Hospital of Cook County, Chicago, USA; 3 Neonatology, Comer Children's Hospital, Chicago, USA; 4 Neonatology, Flushing Hospital Medical Center, Flushing, USA

**Keywords:** term neonate, fetal heart rate, apgar scores, neonatal resuscitation, meconium stained amniotic fluid, electronic fetal monitoring

## Abstract

Objective

To determine if the presence of meconium-stained amniotic fluid (MSAF) by itself or in combination with abnormal fetal heart tracing (FHT) (category II and III) is associated with poor neonatal outcomes in full-term newborns.

Design/methods

This is a retrospective cohort study. Cases included singleton and full-term neonates with MSAF. Cases were compared to matched controls (clear amniotic fluids) for the outcomes. SPSS (IBM SPSS Statistics for Windows, Version 22.0, Armonk, NY, USA) and SAS 9.4 (SAS Institute Inc., Cary, NC, USA) were used for data analysis.

Results

From 5512 deliveries, 210 cases (MSAF group) and 210 matched controls were identified. Cases and controls were similar in most maternal characteristics. Abnormal FHT was present in 43.2% of cases compared to 17.6% of controls (p<0.001). Low Apgar scores (<7) at one and five minutes were more common in the MSAF group (p=0.03 and 0.007, respectively). The neonatal intensive care unit (NICU) admission rate was also higher in the MSAF group (p=0.002). However, the mean hospital stay was similar in both groups (p=0.44). Twenty-two (10.5%) cases required resuscitation at birth compared to six (2.9%) controls (p=0.003). After applying the logistic regression model to adjust for the FHT pattern and Apgar scores at one minute, the association of MSAF with resuscitation lost significance. The factors associated with resuscitation requirement at birth were Apgar score at one minute (adjusted odds ratios (aOR) 4.1; 95% CI 2.8-5.1, p<0.001) and abnormal FHTs (aOR, 0.03; 95% CI 0.004-0.257, p=0.001).

Conclusions

Neonates born with MSAF were more likely to have abnormal FHT and require resuscitation at birth. However, after adjusting for confounding factors, abnormal FHT and one-minute Apgar scores were the only variables predictive of resuscitation needs at birth.

## Introduction

The reported incidence of meconium-stained amniotic fluid (MSAF) varies from 5% in preterm deliveries to 7-22% in term and 23-52% in post-term deliveries [1-4]. MSAF has long been regarded as a sign of fetal distress. However, the mechanism of meconium passage in the fetus is controversial. There are currently two prevailing theories. First, meconium passage could be the result of the normal maturation process of the gastrointestinal tract. This theory is supported by the fact that MSAF is uncommon in preterm deliveries and increases substantially in term and post-term deliveries. Second, it could also be the consequence of pathologic processes, such as hypoxia, increased vagal activity, or infection [5-9].

Irrespective of the etiology, MSAF is associated with adverse neonatal outcomes, including cord acidosis, the need for resuscitation, low Apgar scores, and neonatal intensive care unit (NICU) admission [10-14]. Meconium aspiration before or during labor can result in meconium aspiration syndrome (MAS), which is the most dreaded complication of MSAF. MAS complicates about 2-9% of deliveries with MSAF [1,15]. Until 2015, the neonatal resuscitation program (NRP) recommended routine tracheal suctioning of non-vigorous infants born with MSAF [16]. However, the NRP 2015 guidelines no longer recommend routine endotracheal suctioning in non-vigorous infants delivered through MSAF. The evidence for its efficacy was lacking, and there were more concerns about causing potential harm by continuing this practice [17].

A three-tiered classification of fetal heart rate (FHR) abnormalities has been developed by the American College of Obstetricians and Gynecologists (ACOG), the Eunice Kennedy Shriver National Institute of Child Health and Human Development, and the Society for Maternal-Fetal Medicine [18]. Category I tracing is considered normal and includes a baseline heart rate of between 110 and 160 beats per minute accompanied by moderate variability without any late or variable decelerations. Early decelerations and accelerations are also included in this category [18]. Category II tracings are indeterminate and do not fit into either category I or category III. Category III tracings are considered abnormal and suggest fetal hypoxia and possible acidemia [18,19]. This category includes either no baseline variability or the presence of recurrent late decelerations, variable decelerations, bradycardia, or a sinusoidal pattern [18]. Studies have shown a significant increase in fetal and neonatal morbidity with category III tracing and increased duration in category II tracings. Perinatal morbidity is also associated with a longer duration in category II, specifically the last two hours of labor [19].

In the setting of MSAF with an abnormal FHR tracing, the risk of neonatal morbidity may exceed the risk associated with either factor independently [12-14]. The presence of meconium in amniotic fluid during labor causes apprehension and anxiety to the mothers and alarms the healthcare providers. The relationship between FHT patterns and the outcomes of newborns born with MSAF is still not clear. This study aimed to determine if the presence of MSAF, by itself or in combination with abnormal FHT (category II and III), was associated with poor neonatal outcomes in full-term newborns.

## Materials and methods

This retrospective cohort study was conducted at Flushing Hospital Medical Center, Flushing, New York, from October 2012 to July 2014. The study was approved by the institutional review board (IRB). Term infants born with MSAF (cases) were compared with a matched control group of infants born with clear amniotic fluid for maternal and neonatal characteristics. The inclusion criteria for cases were: gestational age of 37-42 weeks, cephalic presentation, and singleton pregnancy with MSAF.

The very next born infant with the same mode of delivery after each case constituted matched control (1:1) if it also met the following additional criteria: gestational age of 37-42 weeks, cephalic presentation, and singleton pregnancy without MSAF. Infants born with congenital malformations and antenatally diagnosed medical or surgical conditions were excluded from the study.

The diagnosis of MSAF was based on the documentation in the maternal chart. Data for gestational age, parity, group B streptococcus (GBS) infection, pregnancy-induced hypertension (PIH), diabetes, prolonged rupture of membrane (PROM), and mode of delivery were extracted from the electronic medical records. Gestational age was based on the first ultrasound study of the pregnancy or the last menstrual period. Resuscitation was considered present if an infant required positive pressure ventilation for any duration at the time of delivery. Meconium aspiration syndrome was defined as the presence of respiratory distress with typical chest x-ray findings in newborns within the MSAF group. The primary outcome of the study was the requirement for resuscitation at birth. The secondary outcomes were Apgar scores at 1 and 5 minutes, NICU admission, and length of stay (LOS).

Comparisons between the two groups for categorical variables were done with Pearson’s chi-square test (or Fisher’s exact tests, where appropriate). For continuous variables, the student’s t-test or the Mann-Whitney test was used to compare the means or medians. A multivariable logistic regression model was used to investigate the independent contribution of another variable to the outcome. Univariable and multivariable logistic regression analysis was used to calculate the unadjusted and adjusted odds ratios (aOR) with a 95% confidence interval (CI).

The variable of interest was a compound variable defined as a combination of MSAF with abnormal tracing (category II or III). The corresponding sensitivity, specificity, positive predictive value (PPV), and negative predictive value (NPV) were calculated for the variable of interest. A sample size of 82 in each group was obtained with the assumption of a 20% difference in the outcome of resuscitation requirement across the two groups with 80% power and with an alpha error equal to 0.05. We used SAS 9.4 (SAS Institute Inc., Cary, NC, USA) and SPSS (IBM SPSS Statistics for Windows, Version 22.0, Armonk, NY, USA) for our statistical analysis. A P-value of <0.05 was considered significant.

## Results

**Figure 1:**
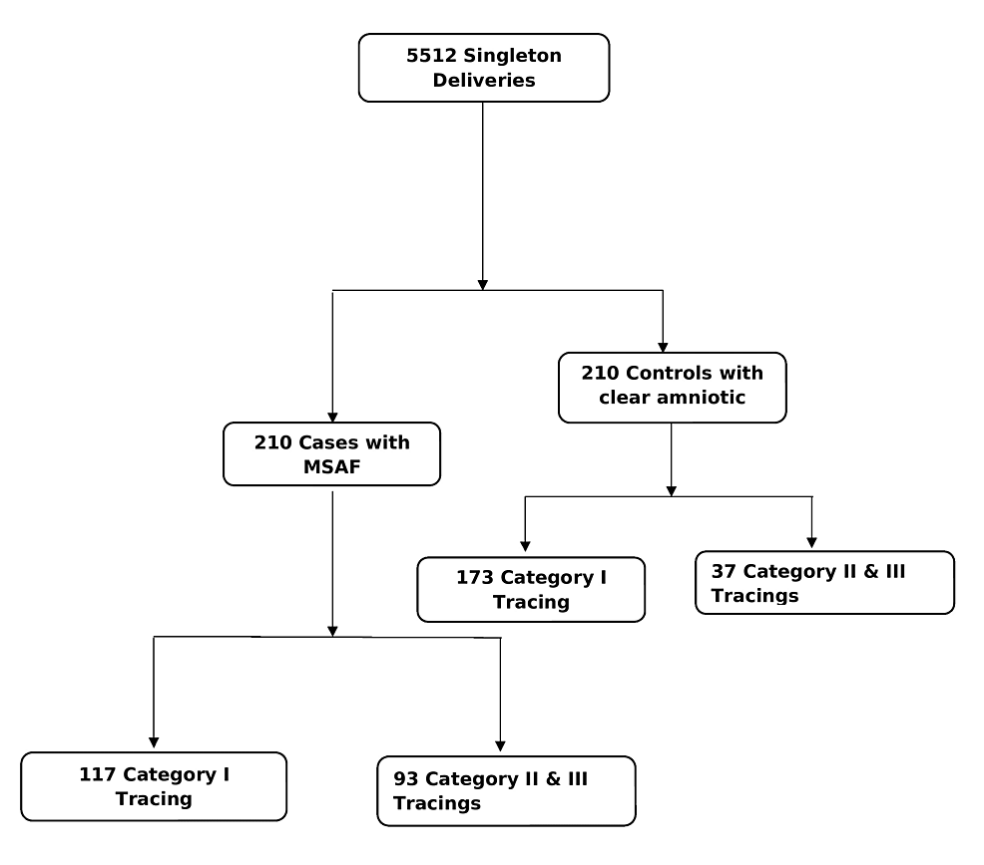
Flowchart showing study population and fetal tracing patterns. MSAF: meconium-stained amniotic fluid.

The total number of qualifying deliveries during the study period was 5512. Out of these, 210 (3.8%) met the inclusion criteria to be included as cases. The same number (210) of controls with clear amniotic fluid were identified from the available pool that met control criteria (Figure 1). Cases and controls were similar for maternal age, nulliparity, male sex, birth weight, gestational age, maternal GBS status, and maternal diabetes (Table 1). Mothers with MSAF were less likely to be diagnosed with PIH (3.8% vs 11.4%; odds ratio (OR) 0.30, 95% confidence interval (CI) 0.14-0.7, P=0.003). The MSAF group had a significantly higher rate of PROM and small for gestational age (SGA) infants than the control group. Table 2 compares outcome characteristics between MSAF and control groups. Infants in the MSAF group were more likely to require resuscitation at birth than infants born with clear amniotic fluid (10.4% vs 2.8%; OR 3.97, 95% CI 1.57-10.03, P=0.002). The NICU admission rate was also higher in the MSAF group compared to the control group (12.3% vs 3.8%; OR 3.57, 95% CI 1.57-8.08, P=0.002). Infants in the MSAF group had a higher rate of low (less than 7) Apgar scores at one and five minutes. However, the mean hospital stay was not different between the two groups (P=0.143).

**Figure 2:**
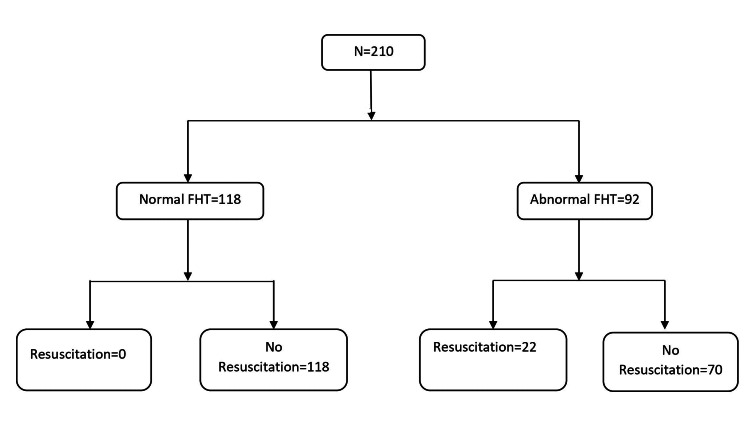
Flowchart showing infants from the MSAF group with fetal tracings and need for resuscitation. FHT: fetal heart tracing and MSAF: meconium-stained amniotic fluid.

Figure 2 shows the outcome of infants with MSAF. An abnormal FHT was present in 93 (44.3%) (85 with category II and 8 with category III) women within the MSAF group compared to 37 (17.6%) (37 with category II and 0 with category III) controls (P<0.001). Category III tracing was significantly higher in cases than in controls (3.8% vs 0% p<0.001). Further, cases were also more likely to have category II tracings than controls (40.5% vs 17.6%; OR 3.39, 95% CI 2.16-5.33). Neonates born with category II fetal tracings were more likely to require resuscitation at birth than infants born with category I tracings (14.8% vs 1.45%; OR 12.37 95% CI 4.1-37.4).

Table 3 compares the characteristics of infants born with normal FHTs (category I) and infants born with abnormal FHTs (category II or category III). Younger mothers were more likely to have abnormal tracings. MSAF, lower one-and five-minute Apgar scores, NICU admission, and length of stay were significantly higher in infants who had abnormal FHTs. The need for resuscitation was also significantly higher in infants with abnormal FHT compared to those with normal tracings. All 22 MSAF infants requiring resuscitation had abnormal FHT, whereas four out of six infants from the control group requiring resuscitation had abnormal FHT. Figure 2 shows infants in both the groups (MSAF and control) with normal and abnormal tracings and their outcomes in terms of resuscitation at birth.

**Table 1:** Comparison of demographic and obstetrics variables among cases and controls. GBS: group B-streptococci, PROM: prolonged rupture of membrane. Values are represented as mean ± standard deviation, N (%), or median (interquartile range). *Fisher exact test and **Mann-Whitney test.

	Cases (N=210)	Controls (N=210)	P-value
Maternal age in years	29.1 ± 5.6	28.5 ± 5.4	0.25
Maternal age >35 years	34	32	0.789
Teen pregnancy	4	16	0.01^*^
Gestational age	39.6 ± 1.1	39.1 ± 1.0	0.62
Birth weight	3227 ± 486	3275 ± 474	0.023
Male gender	95(45.2)	92(43.8)	0.77
Pregnancy-induced hypertension	8(3.8)	24(11.4)	0.003
GBS positivity	12(5.7)	24(11.4)	0.49
Maternal diabetes	28(13.3)	26(12.4)	0.77
PROM	22(10.5)	6(2.8)	0.002
Gravida	2(1.75-3)	3(2-3)	0.043^**^
Gravida >4	10(4.8)	20(5.6)	0.087
Nulliparity	52(24.8)	48(22.8)	0.625
Small for gestational age	8(3.8)	0(0)	0.007
Large for gestational age	11(5.2)	12(5.7)	0.83

**Table 2:** Comparison of outcome characteristics of cases and control group. Values are represented as N (%), or median (interquartile range), *Mann-Whitney test. NICU: neonatal intensive care unit.

	Cases (N=210)	Controls (N=210)	P-value
Apgar 1 min <7	24(11.4)	8(3.8)	0.003
Apgar 5 min <7	24(11.4)	8(3.8)	0.003
Resuscitation	22(10.5)	6(2.8)	0.002
NICU admission	26(12.4)	8(3.8)	0.001
Length of stay	2(2-3)	2(2-3)	0.143^*^
Category I tracing	117(55.7)	173(82.4)	<0.001
Category II tracing	85(40.5)	37(17.6)	<0.001
Category III tracing	8(3.8)	0	<0.001
Abnormal tracing	93(44.3)	37(17.6)	<0.001

**Table 3:** Comparison of characteristics among normal and abnormal fetal heart tracings groups. MSAF: meconium-stained amniotic fluid, PROM: prolonged rupture of membrane, GBS: group-B streptococcus, NICU: neonatal intensive care unit. Values are represented as mean ± standard deviation, N (%), or median (interquartile range). *Fisher exact test and **Mann-Whitney test.

	Normal tracings (N=290)	Abnormal tracing (N=130)	P-value
Mean maternal age in years	29.9 ± 5.7	26.8 ± 4.6	<0.001
Nulliparity	75(25.8)	25(19.2)	0.151
Mean gestation age in weeks	39.3 ± 1.0	39.4 ± 1.1	0.206
Gravidity >4	24	6	0.178
MSAF	117(40)	93(72)	<0.001
PROM	17	11	0.323
GBS positivity	21(11.1%)	15(11.5)	0.146
Pregnancy-induced hypertension	23(7.9)	9(6.9)	0.843
Maternal diabetes	45(15.5)	9(6.9)	0.015
Mean birth weight	3334 ± 446	3289 ± 380	0.158
Large for gestational age	20(6.9)	3(2.3)	0.064^*^
Small for gestational age	6(2.1)	2(1.5)	0.713^*^
Apgar 1 min <7	8(2.8)	24(18.5)	<0.001
Apgar 5 min <7	8(2.8)	24(18.5)	<0.001
Resuscitations	4(1.3)	24(18.5)	<0.001
NICU admissions	8(2.8)	26(20)	<0.001
Length of stay in days	2(2-3)	3(2-3)	0.003^**^

After applying the logistic regression model with resuscitation as an outcome to adjust for FHT pattern, MSAF, and Apgar scores at one minute, the association of MSAF with resuscitation lost significance. Apgar score at one minute (aOR, 4.1; 95% CI 2.8-5.1, p<0.001) and abnormal tracing (aOR, 0.03; 95% CI 0.004-0.257, p=0.001) were the factors significantly associated with the requirement for resuscitation at birth. The presence of MSAF lost its significance for predicting resuscitation.

Moreover, applying the adjusted regression model to the combined variable of interest (combination of MSAF with abnormal tracings) with resuscitation as the outcome, the odds of needing resuscitation were 8.82 times higher in this group compared to the reference group (normal tracing with clear amniotic fluid) (aOR, 8.8); 95% CI 1.3-59.2, p<0.001). The sensitivity, specificity, PPV, and NPV with corresponding 95% CI for the variable of interest in predicting the requirement of resuscitation at birth were 92.3 (82.1-100), 98.33 (96.7-99.9), 85.7 (72.7,98.7), and 99.2 (98-100), respectively.

## Discussion

The rate of MSAF deliveries in our study was 3.8% compared to 7-22% from previously reported studies in term pregnancies [1,2]. This may be in part due to earlier inductions and deliveries and a low threshold for cesarean sections. We also found that PIH was associated with a decreased risk of MSAF. This is again most likely a consequence of earlier induction and deliveries in pregnancies complicated by PIH. We found a significantly increased rate of resuscitation, NICU admission, and lower (<7) one- and five-minute Apgar scores in infants born with MSAF compared to infants born with clear amniotic fluid. This is in agreement with previous studies that have shown increased morbidity in infants born with MSAF [10,11]. Berkus et al. found three times increased risk of adverse neonatal outcomes in infants born with thick meconium [10]. Similarly, Ziadeh et al. and Shaikh et al. also found an increased risk of low Apgar scores, higher NICU admission, as well as neonatal death in neonates born with MSAF [11,20]. However, a few earlier studies had shown no decrease in Apgar scores in pregnancies complicated by MSAF [21-23]. We also noted in our study that the MSAF group had a significantly higher rate of PROM and SGA infants than the control group. This most likely is related to the fact that MSAF is diagnosed more commonly with PROM and gestational advanced neonates.

Our study was conducted in an era when endotracheal suctioning was routinely performed for all non-vigorous MSAF infants. A skilled provider capable of intubating these non-vigorous infants was present in the delivery room/operating room. None of the infants in our study population were diagnosed with MAS. Although multiple studies have been published following the implementation of new NRP guidelines on suctioning for MSAF, the ideal management of non-vigorous MSAF infants is still not clear. Studies by Chettri et al., Nangia et al., Kumar et al., and Myers et al. have shown no significant difference in the outcome of non-vigorous infants managed with and without routine endotracheal suctioning (ETS) [23-26]. At the same time, a study by Singh et al. showed an increased incidence of MAS and prolonged hospital stays for non-vigorous infants without ETS [27]. Similarly, Chiruvolu et al. found an increased incidence of NICU admission for respiratory issues in the non-ETS group of non-vigorous infants [28]. Even though we do not have the data to compare the outcomes of infants post-implementation of NRP 7th edition in our unit, the fact that we had no cases of MAS following ETS in non-vigorous would be an important consideration. It is also important to note that none of these studies have noted worse outcomes in the ETS group compared to no ETS group of non-vigorous infants.

Abnormal FHTs were significantly more common in the MSAF group compared to controls. None of the patients in the control group had category III tracings compared to 3.8% in the MSAF group. Category III tracings are always considered abnormal and most indicate ongoing fetal hypoxia and acidemia [18,19]. The significance of category II tracings is less clear. In our study, category II tracings were more common in the MSAF group. Infants born with category II tracings were more likely to require resuscitation at birth than infants born with category I tracings. Jackson et al. found that increased duration in category II before delivery was associated with an increased rate of low five-minute Apgar scores and NICU admissions [29]. However, the majority of infants born with category II FHR tracing usually have normal outcomes [30].

After applying the logistic regression model with resuscitation as an outcome to adjust for the FHT pattern, MSAF and Apgar scores at one minute, we noted that the association of MSAF with resuscitation had lost significance. Furthermore, abnormal FHR tracings in the presence of MSAF further increase the risk of adverse neonatal outcomes [12-14]. In our study, the PPV and NPV of abnormal FHT in the MSAF group for predicting the need for resuscitation at birth were only 85.7% and 99.2%, respectively. Thus, we may interpret that a normal FHT tracing pattern even in the presence of MSAF will generally predict the normal neonatal outcome. FHTs are routinely monitored in labor and delivery units, and combining the status of FHTs in MSAF deliveries can better help in predicting the need for resuscitation. This may further aid in planning the delivery and deciding if a skilled neonatal provider should attend that delivery in the absence of other risk factors.

One of the limitations of our study was the inability to compare fetal cord gases in the MSAF and control groups, which would have helped in studying the impact of MSAF and abnormal tracing on cord gases. Since most of the control group of infants had no cord gas performed, we would not have been able to compare them between the two groups. The other limitation of our study is the absence of data after the implementation of the 7th edition of NRP. This would have helped us evaluate the impact of avoiding ETS in a non-vigorous group of infants by comparing it with the data presented in this study. Finally, our sample size was relatively small, and a small number of confounders were included within the logistic regression model.

## Conclusions

Among pregnancies complicated by meconium staining of amniotic fluid, combining fetal heart tracing may better aid in predicting neonatal outcomes and thus help in planning the delivery and resuscitation of the newborn. More studies with a larger sample size may further assist in predicting the outcomes of newborns with MSAF.
